# Effectiveness and Limitations of Hand Hygiene Promotion on Decreasing Healthcare–Associated Infections

**DOI:** 10.1371/journal.pone.0027163

**Published:** 2011-11-16

**Authors:** Yee-Chun Chen, Wang-Huei Sheng, Jann-Tay Wang, Shan-Chwen Chang, Hui-Chi Lin, Kuei-Lien Tien, Le-Yin Hsu, Keh-Sung Tsai

**Affiliations:** 1 Center for Infection Control, National Taiwan University Hospital, Taipei, Taiwan; 2 Department of Internal Medicine, National Taiwan University Hospital, Taipei, Taiwan; 3 Department of Medicine, National Taiwan University College of Medicine, Taipei, Taiwan; Columbia University, United States of America

## Abstract

**Background:**

Limited data describe the sustained impact of hand hygiene programs (HHPs) implemented in teaching hospitals, where the burden of healthcare-associated infections (HAIs) is high. We use a quasi-experimental, before and after, study design with prospective hospital-wide surveillance of HAIs to assess the cost effectiveness of HHPs.

**Methods and Findings:**

A 4-year hospital-wide HHP, with particular emphasis on using an alcohol-based hand rub, was implemented in April 2004 at a 2,200-bed teaching hospital in Taiwan. Compliance was measured by direct observation and the use of hand rub products. Poisson regression analyses were employed to evaluate the densities and trends of HAIs during the preintervention (January 1999 to March 2004) and intervention (April 2004 to December 2007) periods. The economic impact was estimated based on a case-control study in Taiwan. We observed 8,420 opportunities for hand hygiene during the study period. Compliance improved from 43.3% in April 2004 to 95.6% in 2007 (*p*<.001), and was closely correlated with increased consumption of the alcohol-based hand rub (*r* = 0.9399). The disease severity score (Charlson comorbidity index) increased (*p* = .002) during the intervention period. Nevertheless, we observed an 8.9% decrease in HAIs and a decline in the occurrence of bloodstream, methicillin-resistant *Staphylococcus aureus*, extensively drug-resistant *Acinetobacter baumannii*, and intensive care unit infections. The intervention had no discernable impact on HAI rates in the hematology/oncology wards. The net benefit of the HHP was US$5,289,364, and the benefit-cost ratio was 23.7 with a 3% discount rate.

**Conclusions:**

Implementation of a HHP reduces preventable HAIs and is cost effective.

## Introduction

Healthcare-associated infections (HAIs) cause significant concern regarding the safety and quality of healthcare quality worldwide [1], [2]. The World Health Organization launched the World Alliance for Patient Safety in October 2004 [2]. HAIs have been identified as a fundamental priority, and were selected as the topic of the first Global Patient Safety Challenge. Hand hygiene was identified as the core component of this strategy because it is a simple, standardized, low-cost measure based on solid scientific evidence. The major barrier is poor compliance by healthcare providers, regardless of available resources [1]–[3].

Following the SARS epidemic in 2003, we reviewed the strengths and weaknesses of infection control strategies at the National Taiwan University Hospital in anticipation of other infectious diseases emerging [4], [5]. We decided to focus first on compliance with hand hygiene. An unannounced hand hygiene audit by infection control nurses was conducted of hospital wards in December 2003. We found that of the 226 opportunities presented, hospital staff washed their hands for only 16.6%. This finding led us to introduce alcohol-based hand rubs, and implement a hospital-wide program promoting hand hygiene through using alcohol-based hand rubs.

Facing an increase in overall HAIs and infections caused by multidrug-resistant organisms [6], the goal of this initiative was to decrease HAIs by blocking the transmission of microorganisms via the hands of healthcare workers (HCW). We used a quasi-experimental, before and after, study design [7] with prospective hospital-wide surveillance of HAIs to assess the effectiveness of hand hygiene. We found that the sustained improvement of hand hygiene compliance reduces HAIs and is cost effective.

## Methods

### Hospital and study population

National Taiwan University Hospital (NTUH) is a 2,200-bed major teaching hospital in Taiwan that provides both primary and tertiary medical care. Approximately one third of the hospital's house staff on the floor are replaced each year. The distribution and time trends of HAIs and infection control programs during 1981 to 2007 have been described previously [6]. An antimicrobial stewardship program was not conducted during the study period.

### Baseline evaluation

We conducted a baseline evaluation in December 2003 [8]. At that time, hands-free washing facilities with unmedicated liquid soap (not refilled) and paper towels were located in every room of the wards and by every intensive care unit (ICU) bed. However, alcohol-based hand rubs were unavailable. The baseline evaluation included direct observation of hand hygiene compliance by infection control nurses, a survey of knowledge and the reasons or factors affecting hand hygiene adherence using a structured, self-administered questionnaire, and suggestions to improve hand hygiene performance. Accordingly, we introduced accessible alcohol-based hand rubs to improve compliance.

### Hospital-wide hand hygiene program

With substantial support from the hospital superintendent, the hospital-wide hand hygiene promotion program began in April 2004. A multidisciplinary approach involving cognition, equipment, and behavior was designed based on literature, the baseline evaluation, and the concept of total quality improvement [9]. Disposable alcohol-based hand rub sanitizers (75% isopropyl alcohol in plastic hand-compressing dispensers) (So Easy liquid, PBF, Taiwan) were wall mounted between every two beds in general wards, by each bed in special units (such as intensive care units), and affixed to trolleys (including treatment trolleys and resuscitation trolleys) to ensure accessibility near or at the point of care [2].

Promotion consisted of lectures and/or web-based self-learning with a post test, reminders located near points of care, use of hand hygiene compliance as a quality indicator, observation and verbal reminders by infection control nurses, periodic audits and performance feedback provided to units and departments, and incentives of US$160.00 for an outstanding performance (unit and department level). A fine of US$3.00 for compliance failures (individual level) was implemented in 2007 in highly specific situations, that is, individuals not modify their behavior even after face-to-face communication. The hand hygiene program was announced and promoted hospital-wide for one month every year while preparing for pandemic influenza (typically during November). The program was reviewed, revised, and promoted annually according to the plan-do-check-act cycle [8]. The target hand hygiene compliance rate was determined based on the performance in the preceding year.

### Determination of hand hygiene compliance

**Figure 1:**
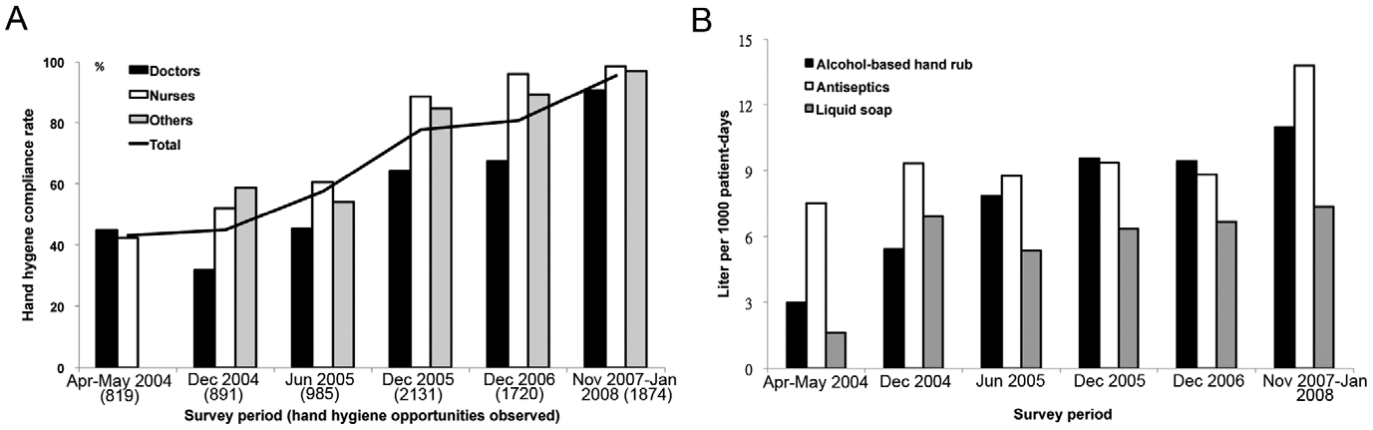
Trends in compliance with the hand hygiene during 6 consecutive hospital-wide surveys conducted from May 2004 to December 2007. Panel A shows significant increases in percent of adherence to hand hygiene before and after patient contact for all healthcare workers in in-patient service units (*p*<0.001), by doctors (*p*<0.001), nurses (*p*<0.001), and other healthcare workers (*p*<0.001). Panel B shows significant increases in the annual consumption of alcohol-based hand rub (*p* = 0.001), antiseptics (*p* = 0.04), and liquid soap (*p* = 0.03).

Hand hygiene compliance was assessed by direct observation during day shifts (8 a.m. to 5 p.m.) on weekdays according to the U.S.A. CDC criteria [10], using a standardized case report form. Emergency procedures were excluded. Direct observation was performed by infection control nurses (ICN) after training and consensus development [11]. The site audit period was announced, though healthcare workers were not informed of the specific observation time. The hand hygiene compliance rates recorded for comparison between hospital services or between years were limited to the opportunities before and after patient contact to maintain consistency during the study period. Six surveys were conducted during the implementation period. To avoid the Hawthorne effect [12], the duration and number of opportunities for hand hygiene monitoring were increased gradually for the six surveys (Fig. 1A). The annual consumption of liquid soap, antiseptics, and alcohol hand rub (product volume use per 1,000 patient-days) was used as a surrogate marker of hand hygiene over time.

### Outcome assessment and data collection

We measured the overall HAI rates, HAI rates by site of infection, and HAI rates by selected pathogen to assess the effectiveness of HHP. We chose methicillin-resistant *Staphylococcus aureus* (MRSA), *Acinetobacter*, and extensively drug-resistant *A. baumannii* (XDRAB) as marker organisms because our previous study showed a significant increase in HAI incidences, and outbreaks of these infections occurred during the study period [6]. Conversely, the incidences of HAIs by methicillin-susceptible *S. aureus* (MSSA) and *Escherichia coli*, which were chosen as control organisms, were relatively stable or decreased.

Prospective, hospital-wide on-site surveillance of HAIs at NTUH began in 1981, and were conducted through weekly visits from full-time ICN to all patient units [6]. Data were collected on standardized data collection forms and inputted into the computer database manually. The severity of underlying diseases, comprising a maximum of six diseases, was scored using the Charlson comorbidity index [13]. The patient population, bed occupancy rate, age, gender, severity of underlying diseases, length of hospital stay, cumulative incidence of HAI, and outcome at discharge were determined during the preintervention (January 1999 to March 2004) and intervention (April 2004 to December 2007) periods.

### Definitions

HAIs were classified according to definitions provided by the Centers for Disease Control and Prevention, U.S.A. [6], [14]. The cumulative incidence of HAIs was defined as episodes per 1,000 patient-days. Extensively drug-resistant *A. baumannii* (XDRAB) was defined as isolates that were resistant to five or more classes of antibacterial agents [6]. Compliance with hand hygiene is the ratio of the number of performed actions using correct technique to the number of opportunities [2], [11].

### Statistical evaluation

To investigate the impact of the intervention on the levels and trends of cumulative HAI incidence over time, we adopted the generalized autoregressive Poisson regression analyses [15], [16]. This model autocorrelates the HAI cases in successive periods by incorporating the autoregressive order *j* term and number of HAI cases at an earlier time *t*-*j* [17]. The factors considered by the model (see Text S1) include the levels and trends in the preintervention period, the changes in levels and trends during the intervention period, and autoregressive terms. Because the SARS epidemic occurred during the later part of the preintervention period (April 2003 to July 2003), an additional variable was added to the model to assess the effect of SARS. To account for possible seasonal and other event variations, such as the lengthy Chinese New Year holiday during January and February, the “old guy” effect during April and May, and the new staff effect during June to July, the model defined spring as March to May, summer as June to August, autumn as September to November, and winter as December to February. The analysis was further stratified by the site of infection, such as bloodstream, urinary tract, and surgical site infections, by the pathogens, such as MSSA, MRSA, *Acinetobacter*, XDRAB, and *E. coli*, and by ward units, such as ICUs, oncology wards, and hematology wards. Heterogeneity factors were calculated by dividing the deviance with the degrees of freedom, and used as an indicator to assess whether an extra-Poisson variation (overdispersion) was present.

The difference in patient population during the two periods was examined using Student's *t*-test (continuous variables) and a chi-squared test (categorical variables). Time trends in the consumption of alcohol-based hand rubs, soap, and antiseptics were examined using simple linear regression. To investigate improvements to hand hygiene compliance during the six consecutive hospital-wide surveys, Pearson's chi-squared test for trend was performed. Correlation between hand hygiene compliance and the consumption of alcohol-based hand rubs, soap, and antiseptics, was examined using the Spearman method. All statistical tests were considered two-tailed and were significant at *p*<.05.

### Economic evaluation

The relevant parameters used in the economic evaluation are shown in Table S2. The cost-effectiveness of the program was evaluated by calculating the extra cost required to prevent one episode of HAI from a hospital perspective. The number of expected episodes of HAI averted by the program was derived using the generalized autoregressive Poisson regression model. The costs considered in the analysis included alcohol-based hand hygiene products and promotional efforts (posters, wall displays, rewards, and other expenses) over the 4-year intervention period. The cost of personnel involved in the program (including planning, training, and auditing) was not considered in the base-case analysis (Table S2), because the program did not incur any increases in staff or manpower costs. However, the opportunity costs of personnel were considered in the sensitivity analysis by converting the number of working hours that infection control nurses spent on the program to their salary (Table S3). Cost-benefit analyses were also conducted to examine the net benefit and benefit-cost ratio of the program. The benefit was measured by subtracting the cost savings from the extra costs caused by HAIs, which was determined by a case-control study conducted in our hospital [18]. All future costs, number of HAI episodes, and benefits were discounted to the present value at an annual rate of 3% [19], [20]. One-way sensitivity analyses were performed to explore the influence of the uncertainty of several parameters, such as discount rates, cost of alcohol hand rub, campaign expenses, extra cost per HAI episode, and the number of averted HAIs. The ranges used for the sensitivity analysis were a 50% increase or decrease to the base-case estimates of alcohol hand rub costs and campaign expenses, the twenty-fifth and seventy-fifth percentile of the estimated additional costs of each HAI episode, and a 95% confidence interval for the model-predicted number of averted HAIs.

### Ethics statement

We followed the principles expressed in the Declaration of Helsinki. This study was approved by the Institutional Ethics Review Board of the National Taiwan University Hospital (No. NTUH-200805033R).

## Results

### Patient population

The patient population characteristics during the preintervention and intervention periods are summarized in Table 1. During the intervention period, the total patient days (*p* = .004), mean Charlson comorbidity index (*p* = .002), and frequency of underlying illnesses, such as hematological malignancies and solid tumors (*p*<.001), increased significantly.

**Table 1:** Characteristics of the Patient Population.

Parameter	Preintervention period (Jan 1999–Mar 2004)	Intervention period (April 2004–Dec 2007)
Number of acute care beds	2,027±180	2,202±8.6
Occupancy rate, %	79.7±6.7	84.1±0.9^a^
Total patient-days	648,305±32,109	720,564±9,543^b^
Length of hospital stay (days)	9.80±0.62	9.79±0.65
Number of patients	290,056	262,090
Age (in years)	46.4±23.1	48.3±23.3^a^
Gender, % male	50.4±1.2	49.2±1.3
Charlson comorbidity index	2.07±0.27	2.81±0.22^c^
Underlying diseases, %		
Cardiac vascular diseases	16.6±3.2	22.9±0.3^a^
Congestive heart failure	1.8±0.3	2.4±0.1^a^
Cerebrovascular diseases	4.4±0.8	4.8±0.3^a^
Chronic pulmonary diseases	3.5±0.8	4.1±0.3^a^
Moderately-severe liver diseases	5.9±0.8	3.8±0.1^a^
Moderately-severe renal diseases	4.8±0.8	3.6±0.5^a^
Diabetes mellitus without end organ damage	7.4±1.5	9.8±0.2^a^
Diabetes mellitus with end organ damage	1.5±0.2	1.6±0.1
Any tumor	22.0±1.7	31.7±3.3^a^
Leukemia	0.37±0.06	0.47±0.03^a^
Lymphoma	1.10±0.25	1.63±0.16^a^
Metastatic solid tumor	7.53±1.16	12.80±2.01^a^
Acquired immunodeficiency syndrome	0.37±0.07	0.40±0.02
Solid organ transplant (kidney, liver, heart, or pancreas)	0.59±0.06	0.93±0.14^a^
Hematopoietic stem cell transplant	0.05±0.01	0.05±0.02
In-hospital mortality, %	3.67±0.19	3.77±0.09^d^

a *P*<0.001.

b *P* = 0.004.

c *P* = 0.002.

d *P* = 0.04.

### Hand hygiene compliance

During the six surveys conducted during the intervention period, 8,420 opportunities to observe hand hygiene were presented, as shown in Fig. 1A. Overall compliance improved from 43.3% in 2004 to 95.6% in 2007 (*p*<.001). Compliance improved significantly for all professional categories of HCW (*p*<.001), in both general wards and intensive care units (*p*<.001). We also observed an increased use of alcohol-based hand rubs (*p* = .001), liquid soap (*p* = .03), and antiseptics (*p* = 0.04), as shown in Fig. 1B. The overall improvement in hand hygiene compliance was significantly correlated with the increased consumption of alcohol-based hand rub (correlation coefficient *r* = 0.9399, *p* = .005), but less correlated with the consumption of antiseptics (*r* = 0.7930, *p* = .06) and soap (*r* = 0.7686, *p* = .07).

### Outcome assessment

**Figure 2:**
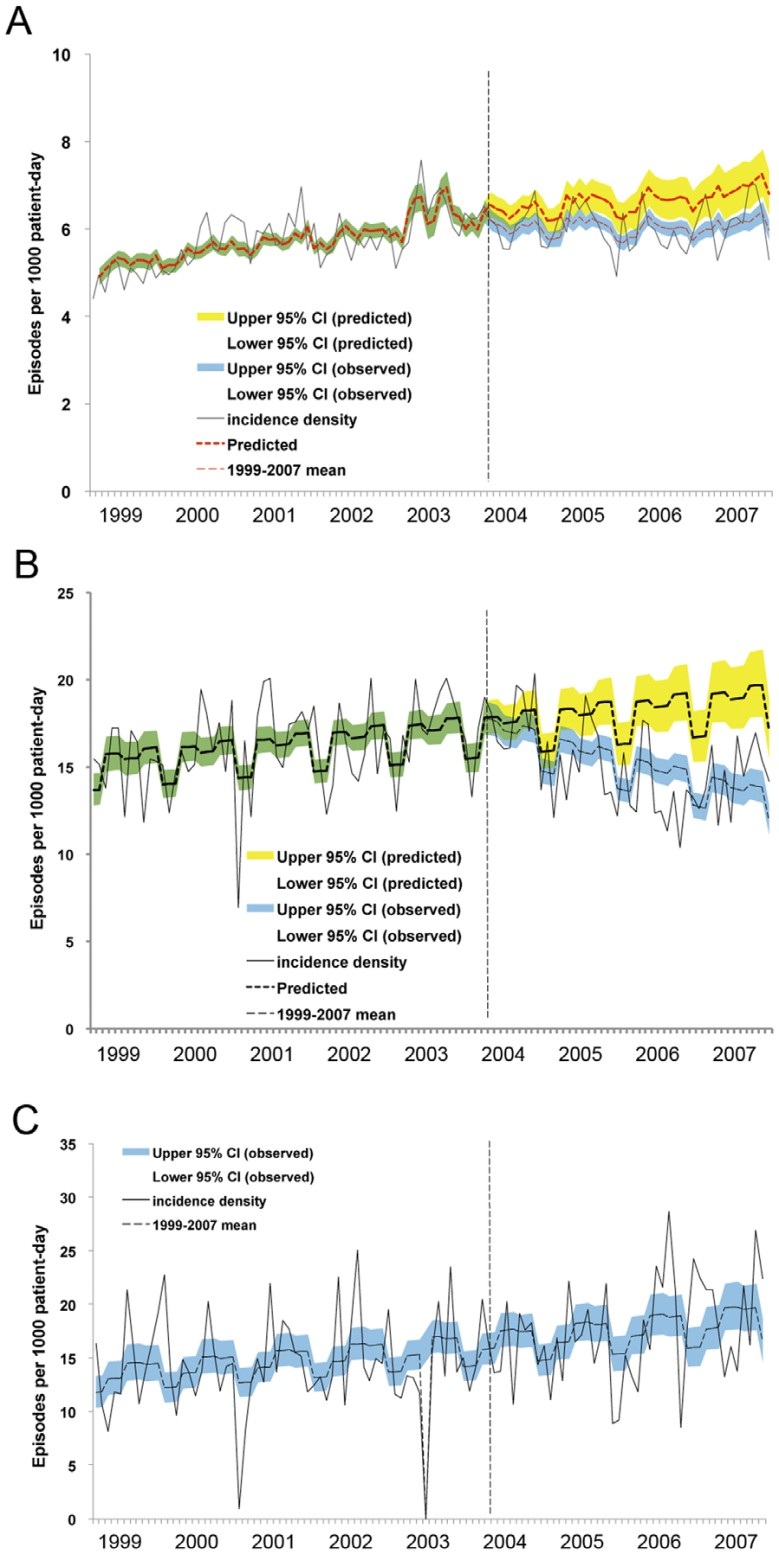
Time trends of monthly cumulative incidences of overall healthcare-associated infection before (January 1999 to March 2004) and during the hand hygiene program (April 2004 through December 2007). (A) hospital-wide (change in levels, *p* = 0.02; change in trends, *p* = 0.04); (B) intensive care units (change in levels, *p* = 0.26; change in trends, *p*<0.001); (C) hematology ward (*p* = 0.21, *p* = 0.38, respectively). Observed incidences, black solid line, —; mean, red dash line, ---; green and blue shadow, 95% confidence interval of observed incidences; yellow shadow, 95% confidence interval (CI) of predicted incidences. The vertical dashed lines (- - -) separate the preintervention and intervention periods.

The predicted monthly cumulative incidence of HAI using the full and most parsimonious segmented regression models are shown in Table S1. Before intervention, HAI increased gradually, and the impact of SARS and seasons were significant. After implementation of the hand hygiene program, the levels and trends of HAIs changed significantly (*p* = .02, *p* = .04, respectively), as shown in Fig. 2A. Bloodstream (*p<*.001), urinary tract (trend, *p* = .03), and skin and soft tissue infections (trend, *p*<.001) decreased significantly. Additionally, though surgical site infections showed a downward trend, the incidence rate did not differ significantly. No significant changes in the respiratory tract and gastrointestinal tract infection rates were observed during the intervention. The time trends of HAI significantly decreased in ICUs (*p*<.001) during the intervention period, as shown in Fig. 2B; however, no significant changes in the HAI rates of oncology and hematological wards was noted, as shown in Fig. 2C.

**Figure 3:**
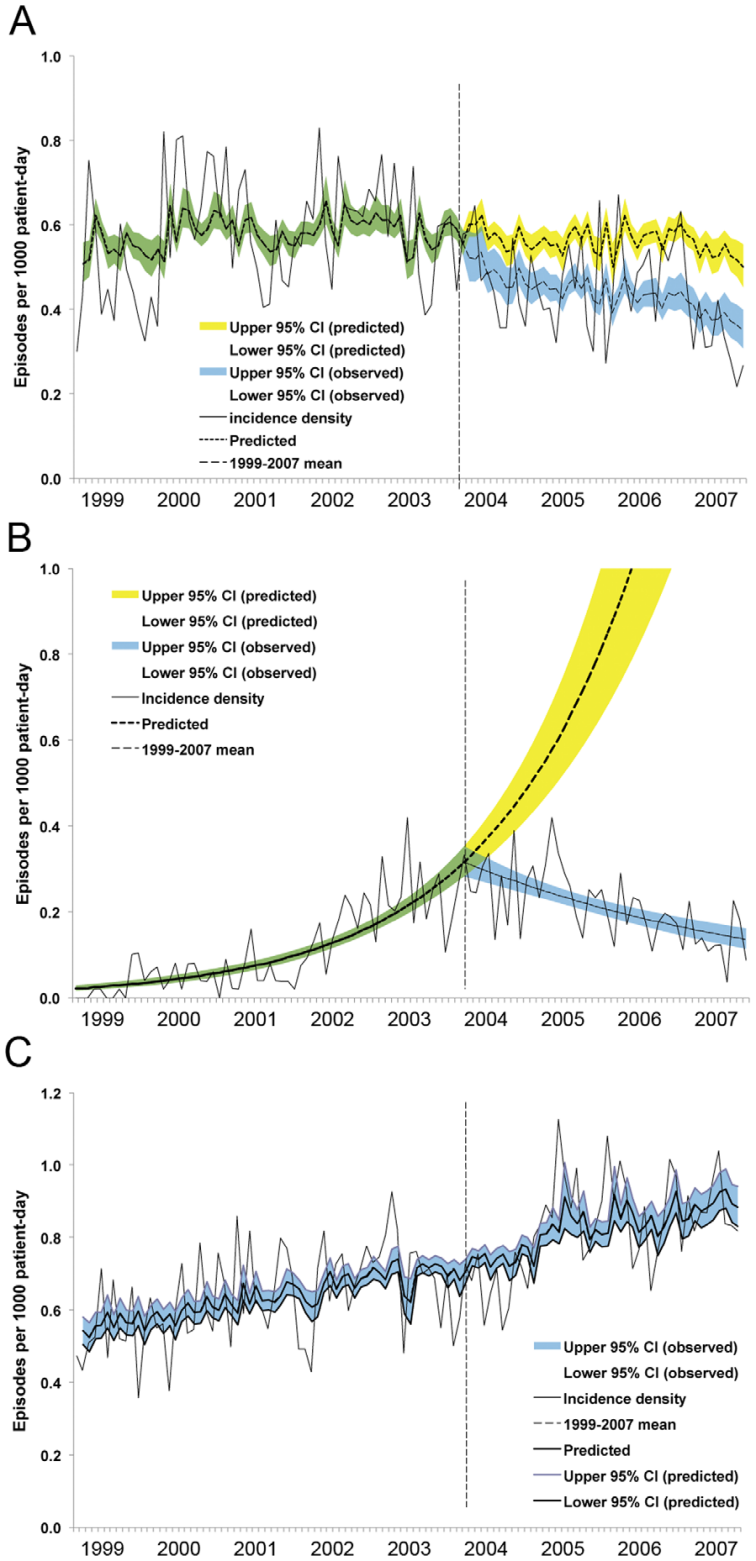
Time trends of monthly cumulative incidences by pathogen. (A) methicillin-resistant *S. aureus* (MRSA) (change in level, *p* = 0.03; change in trend, *p* = 0.04); (B) extensively drug-resistant *Acinetobacter* (XDRAB) (*p* = 0.78; *p*<0.001, respectively); (C) *Escherichia coli* (*p* = 0.89; *p* = 0.33, respectively). Infection control measures for XDRAB were intensified during June 2001 to June 2002. These efforts resulted in only a transient reduction in the rates of infection for XDRAB and MRSA. Observed incidences, black solid line, —; mean, red dash line, ---; green and blue shadow, 95% confidence interval of observed incidences; yellow shadow, 95% confidence interval (CI) of predicted incidences. The vertical dashed lines (- - -) separate the preintervention and intervention periods.

The cumulative incidence of HAIs caused by MRSA, *Acinetobacter*, and XDRAB decreased substantially during the intervention period, as shown in Figs. 3A and 3B. This was associated with a decrease in the annual consumption of glycopeptides, anti-*Pseudomonas* fluoroquinolones, and carbapenems (data not shown). Finally, no significant change in the trends or levels of infections caused by MSSA and *E. coli* during the intervention period was observed, as shown in Fig. 3C.

### Economic evaluation

The hand hygiene program was associated with an estimated reduction of 1,504 (95% confidence interval: 526 to 2,544) HAI episodes (8.9%) during the intervention period without discounting (Table 2). Results of the economic analysis with a 3% discount rate are shown in Table 3. From a hospital perspective, the discounted additional cost of preventing one HAI episode was US$163.6. However, the net benefit of the hand hygiene program is US$5,289,364, which indicates that the benefits from savings to the additional costs of HAI could outweigh the program costs. The benefit-cost ratio shows that every US$1 spent on the program could result in a US$23.7 benefit. The results of one-way sensitivity analysis (Table S3) show that the cost of alcohol hand rub and the number of averted HAI episodes are the two factors influencing the additional cost of preventing one HAI episode; extra cost per HAI episode and the number of averted HAI episodes significantly impact the net benefit and benefit-cost ratio.

**Table 2:** Decrease in Healthcare-associated Infections and Cost Savings Attributed to the Hand Hygiene Program.

Parameter	Number
Episodes of healthcare-associated infections	
Observed	15,301
Predicted^a^	16,805
Total reduction (%)	1,504 (8.9%)
Costs of the hand hygiene program in US dollars	
Total alcohol handrub expense	221,517^b^
Campaign costs (posters, salaries,^c^ etc.)	22,953
Total cost^d^	244,470
Average cost per 1000 patient-day	90.60
Average cost to prevent one episode of HAI	162.50
Extra costs per episode of HAI^e^	5,335±13,872
Mean cost reduction for HAIs	8,023,840
Mean net cost savings from the hand hygiene program	7,779,370

a Predicted by Poisson regression model described in Methods and Text S1.

b All monetary values are expressed in US dollars. The average exchange rate in 2007: 1 US dollar = 32.842 New Taiwan dollars, 1 Euro = 44.952 New Taiwan dollars.

c The salaries were limited to the proportion of working hours of infection control nurses spent for the hand hygiene audits, not include those of infection control nurses in planning and education and not include those of infectious disease specialists.

d Variable costs including soap, water and materials used for drying hands (e.g., towels) were not included.^8^

e Based on a case-control study conducted at this hospital and two local hospitals in Taiwan.^18^

**Table 3:** Results of Cost-effectiveness Analysis and Cost-benefit Analysis of the Hand Hygiene Program^a^.

Parameter	Value
Episodes of healthcare-associated infections^b^	
With hand hygiene program	14,608
Without hand hygiene program	16,032
Number of averted episodes of HAI	1,424
Saving from extra costs arose from episodes of HAI	5,522,408^c^
Extra costs of the hand hygiene program	233,044
Extra cost to prevent one episode of HAI	163.6
Net benefit^d^	5,289,364
Benefit cost ratio^e^	23.7

a All future costs, number of episodes of HAI, and benefits were discounted to the present value at an annual rate of 3%.

b Predicted by Poisson regression model described in Methods and Text S1.

c All monetary values are expressed in US dollars. The average exchange rate in 2007: 1 US dollar = 32.842 New Taiwan dollars, 1 Euro = 44.952 New Taiwan dollars.

d Net benefit = Benefit – Cost.

e Benefit cost ratio = benefit/cost.

## Discussion

This study demonstrates that excellent compliance with hand hygiene by HCW was achieved and maintained over 4 years through an intensive hospital-wide program. The program was associated with significant decreasing trends for all HAIs and HAIs caused by MRSA and XDRAB, and was cost effective. However, we were unable to demonstrate a reduction in the HAIs of hematology and oncology services. We were also unable to demonstrate a reduction in HAIs caused by MSSA and *E. coli*. This result was unsurprising because of the relatively greater importance of endogenous infections in immune and structurally compromised hosts.

To provide consistent care and protect patient safety throughout the hospital, a hand hygiene program requires multidisciplinary efforts and encompasses all hospital units. However, conducting a randomized, controlled trial in such a large and complex situation is difficult. According to a recent, comprehensive review of literature published as a Cochrane Review [21], the quality of intervention studies intended to increase hand hygiene compliance remains disappointing. We chose to superimpose the hand hygiene program on a well-established infection control program using standardized surveillance methods. This enabled us to determine the differences in HAI incidences over 4-year preintervention and postintervention periods, and conduct numerous observations while adjusting to changes in the frequency and severity of underlying diseases.

We elected to use Poisson regression analysis with an interrupted time series [22], [23] to assess the efficacy of the hospital-wide hand hygiene intervention over time, and determine whether factors other than the intervention could explain the change. In the interrupted time series, the level and trend of the preintervention segment served as the controls for the postintervention segment, providing a methodologically acceptable design for measuring the intervention effect [22], [23]. This method requires data of the continuous or counted outcome measures, summarized at regular, evenly spaced intervals. Thus, evaluation of the longitudinal effect of a hospital-wide hand hygiene program is only feasible in limited healthcare settings.

Although evidence exists that improved adherence to hand hygiene is linked to reduced infection rates [24]–[26], other studies failed to report these effects [27]–[30]. In addition, the impact of hand hygiene improvement on HAI incidence varied [31], [32]. Several potentially confounding factors are relevant to this study. First, although direct observation is the criterion standard for measuring HH compliance, the method is subject to observation bias, selection bias, and the Hawthorne effect [2], [12], which may result in an overestimated HH rate. Second, the HH compliance rate was limited to the opportunities before and after patient contact to maintain consistency during the study period. Our recent survey demonstrated that the HH rate for 5 moments was only approximately 60%; whereas the HH rate for two moments exceeded 90% (unpublished data). Third, the hand hygiene programs in this study focused only on HCWs. Patients, patients' family, and other caregivers may contaminate the environment and/or transfer organisms. Fourth, not all HAIs of exogenous origin can be prevented by hand hygiene [33], [34]. Augmenting other infection control measures, such as multimodal implementation strategies (bundle care), environment cleanliness, appropriate use of antimicrobial agents, and active microbial surveillance of multidrug-resistant organisms is also necessary [33]–[38]. Finally, not all HAI were preventable. Our data failed to support the effects of HHP on decreasing HAIs among hemato-oncology patients and patients with infections caused by MSSA and *E. coli*. Endogenous infection may be reduced through improvements in host immunity.

The economic impact of effective hand hygiene programs on decreasing HAIs was first evaluated by Pittet et al. [2], [24]. Pittet and colleagues [24] estimated the program costs to be less than US$57,000 per year for a 2,600-bed hospital, an average of US$1.42 per admitted patient. Supplementary costs associated with the increased use of alcohol-based hand rub averaged US$6.07 per 100 patient-days. Based on conservative estimates, US$100 was saved per averted infection. The economic evaluation in this study produced similar results. In addition, this study showed that the cost of alcohol hand rub and the number of averted HAI episodes are the two factors influencing the additional costs of preventing one HAI episode, which subsequently influences the net benefit and benefit-cost ratio. However, in the model used in this study, the cost of manpower was underestimated, and the indirect costs related to HAIs were not included. Several studies have demonstrated a temporal relationship between improved hand hygiene practices and a reduction in HAI incidence and multidrug resistant microorganisms [2], [25]; however, few have been able to sustain a lasting impact [2], [26]. Demonstrating to hospital administrators that these programs are cost-effective, relatively inexpensive, improve patient safety, and require long-term and stable investment is essential [2], [24]. This is particularly required by teaching hospitals where the house staff and attending physicians change periodically [25]. Interest in pay-for-performance and other funding schemes is growing, which should further strengthen financial incentives to foster hand hygiene [39]–[41].

In conclusion, this study demonstrates that implementing a hospital-wide hand hygiene program is feasible. The program was associated with a reduction in the HAIs of most hospital units and HAIs caused by MRSA and XDRAB. This effect was achieved and confirmed using a before and after study design combined with a prospective HAI surveillance program and hospital-wide annual promotion, which resulted in sustained effects and high-quality HH observation. The costs of implementing the program were low compared to the costs saved by reducing the HAI incidence. The impact may be even greater with full adherence to the five moments for hand hygiene. However, hand hygiene programs are only one component of hospital infection control. They must be supplemented with measures directed at device-associated and endogenous infections in compromised hosts, and augmented by antimicrobial stewardship.

## Supporting Information

Text S1 **The generalized autoregressive Poisson model.**
(DOC)

Table S1 **Parameter estimates, standard errors and** ***p*** **values from the full and most parsimonious segmented regression models predicting monthly incidence density (episodes per 1000 patient-days) over time.**
(DOC)

Table S2 **Base-case estimates and ranges used in sensitivity analyses of parameters.**
(DOC)

Table S3 **Results of one-way sensitivity analysis.**
(DOC)
