# Supplementary material for: Effectiveness and Limitations of Hand Hygiene Promotion on Decreasing Healthcare–Associated Infections
Source: PLoS One. 2011 Nov 16;6(11):e27163. doi: 10.1371/journal.pone.0027163 (PMC3217962; doi:10.1371/journal.pone.0027163)
Supplement: Text S1 — The generalized autoregressive Poisson model. (DOC) [file pone.0027163.s001.doc]

**Text S1. The generalized autoregressive Poisson model**

The generalized autoregressive Poisson model form is written as Log (*E*(*y_t_*))＝log(PY) + *β_0_* + *β_1_* × *time* + *β_2_* × *SARS* + *β_3_* × *intervention* + *β_4_* × *time after intervention* + *β_5_* × *spring* + *β_6_* × *summer* + *β_7_* × *autumn* + $\sum_{j=1}^{l} \gamma_{j}y_{t-j}$, where *y_t_* is the monthly number of healthcare-associated infection (HAIs) cases at time *t*, PY is the person-years (offset), *β_0_* represents the baseline level of infection in preintervention segment, *time* indicates the linear trend of HAIs and was denoted as time in month from the start of the study period, *SARS* and *intervention* are indicators for the period of SARS epidemic and postintervention segment, respectively, *time after intervention* was denoted as the time in month after the start of intervention and is coded as zero before the intervention to represent the change in trend after intervention, *spring*, *summer*, and *autumn* are the dummy variables for seasonal pattern which takes winter as comparison group, *y_t-j_* is the number of HAIs cases at time *t-j* to represent the autoregressive order *j*, *β_1_*,…, *β_7_*, and *γ_j_* are the regression coefficients for the corresponding variables and can be interpreted as the logarithm of the relative rate (RR). Based on this equation, the effect of intervention can be expressed as $\left(1-\exp\left(\beta_{3}\right)\right)\times100$ for relative change in level of HAIs density, and $\left(1-\exp\left(\beta_{4}\right)\right)\times100$ for relative change in trend of HAIs density
