# Supplementary material for: Effectiveness and Limitations of Hand Hygiene Promotion on Decreasing Healthcare–Associated Infections
Source: PLoS One. 2011 Nov 16;6(11):e27163. doi: 10.1371/journal.pone.0027163 (PMC3217962; doi:10.1371/journal.pone.0027163)
Supplement: Table S1 — Parameter estimates, standard errors and p values from the full and most parsimonious segmented regression models predicting monthly incidence density (episodes per 1000 patient-days) over time. (DOC) [file pone.0027163.s002.doc]

**Table S1. Parameter estimates, standard errors and *p* values from the full and most parsimonious segmented regression models predicting monthly incidence density (episodes per 1000 patient-days) over time**

| **Model** | **Full model** | | | **Parsimonious model** | | |
| --- | --- | --- | --- | --- | --- | --- |
|  | **β** | **95% CI** | ***P*-value** | **β** | **95% CI** | ***P*-value** |
| **Overall infection** |  |  |  |  |  |  |
| Intercept β_0_ | -5.4683 | -5.5831,-5.3536 | <.0001 | -5.4604 | -5.5479,-5.3729 | <.0001 |
| Baseline trend β_1_ | 0.0021 | 0.0008,0.0034 | 0.0011 | 0.0021 | 0.0010,0.0033 | 0.0002 |
| SARS β_2_ | 0.0838 | 0.0284,0.1392 | 0.0030 | 0.0823 | 0.0298,0.1349 | 0.0021 |
| Level change after hand hygiene β_3_ | -0.0544 | -0.1016,-0.0073 | 0.0236 | -0.0539 | -0.1008,-0.0071 | 0.0239 |
| Trend change after hand hygiene β_4_ | -0.0017 | -0.0035,0.0001 | 0.0680 | -0.0017 | -0.0034,-0.0001 | 0.0425 |
| Number of infection at time lag1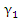 | 0.0007 | 0.0003,0.0010 | 0.0002 | 0.0007 | 0.0003,0.0010 | 0.0001 |
| Season |  |  | 0.0457 |  |  | 0.0430 |
| Season1 (Mar-May) β_5_ | 0.0449 | 0.0116,0.0781 | 0.0082 | 0.0439 | 0.0118,0.0760 | 0.0073 |
| Season2 (Jun-Aug) β_6_ | 0.0331 | 0.0005,0.0657 | 0.0469 | 0.0325 | 0.0004,0.0647 | 0.0474 |
| Season3 (Sept-Nov) β_7_ | 0.0353 | 0.0034,0.0673 | 0.0304 | 0.0353 | 0.0035,0.0670 | 0.0295 |
| Season4 (Dec-Feb) | 0.0000 | **-** | - | 0.0000 | **-** | - |
| **Bloodstream infection** |  |  |  |  |  |  |
| Intercept β_0_ | -6.5543 | -6.6946,-6.4139 | <.0001 | -6.5714 | -6.7089,-6.4339 | <.0001 |
| Baseline trend β_1_ | 0.0036 | 0.0015,0.0057 | 0.0010 | 0.0026 | 0.0011,0.0042 | 0.0006 |
| SARS β_2_ | 0.1007 | 0.0148,0.1865 | 0.0215 | 0.1201 | 0.0402,0.2001 | 0.0032 |
| Level change after hand hygiene β_3_ | -0.1371 | -0.2137,-0.0606 | 0.0004 | -0.1362 | -0.2131,-0.0594 | 0.0005 |
| Trend change after hand hygiene β_4_ | -0.0017 | -0.0045,0.0010 | 0.2230 | **-** | **-** | **-** |
| Number of infection at time lag1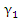 | 0.0012 | 0.0001,0.0024 | 0.0337 | 0.0014 | 0.0003,0.0025 | 0.0123 |
| Number of infection at time lag2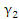 | 0.0013 | 0.0001,0.0025 | 0.0348 | 0.0016 | 0.0004,0.0027 | 0.0072 |
| Season |  |  | 0.0116 |  |  | 0.0182 |
| Season1 (Mar-May) β_5_ | 0.0089 | -0.0463,0.0641 | 0.7523 | 0.0119 | -0.0431,0.0669 | 0.6726 |
| Season2 (Jun-Aug) β_6_ | 0.0594 | 0.0063,0.1126 | 0.0284 | 0.0599 | 0.0067,0.1130 | 0.0273 |
| Season3 (Sept-Nov) β_7_ | 0.0712 | 0.0202,0.1222 | 0.0062 | 0.0668 | 0.0163,0.1173 | 0.0096 |
| Season4 (Dec-Feb) | 0.0000 | **-** | - | 0.0000 | **-** | **-** |
| **Urinary tract infection** |  |  |  |  |  |  |
| Intercept β_0_ | -6.8332 | -6.9653,-6.7012 | <.0001 | -6.8031 | -6.9083,-6.6980 | <.0001 |
| Baseline trend β_1_ | 0.0047 | 0.0025,0.0070 | <.0001 | 0.0039 | 0.0021,0.0058 | <.0001 |
| SARS β_2_ | 0.0799 | -0.0160,0.1757 | 0.1024 | 0.1033 | 0.0160,0.1905 | 0.0203 |
| Level change after hand hygiene β_3_ | -0.0464 | -0.1322,0.0394 | 0.2892 | - | - | - |
| Trend change after hand hygiene β_4_ | -0.0033 | -0.0064,-0.0003 | 0.0307 | -0.0032 | -0.0061,-0.0003 | 0.0321 |
| Number of infection at time lag1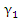 | 0.0028 | 0.0012,0.0044 | 0.0006 | 0.0026 | 0.0011,0.0041 | 0.0006 |
| Season |  |  | 0.0100 |  |  | 0.0129 |
| Season1 (Mar-May) β_5_ | 0.0756 | 0.0170,0.1342 | 0.0114 | 0.0658 | 0.0090,0.1227 | 0.0232 |
| Season2 (Jun-Aug) β_6_ | -0.0118 | -0.0711,0.0475 | 0.6959 | -0.0219 | -0.0798,0.0360 | 0.4585 |
| Season3 (Sept-Nov) β_7_ | 0.0316 | -0.0258,0.0889 | 0.2807 | 0.0258 | -0.0310,0.0826 | 0.3736 |
| Season4 (Dec-Feb) | 0.0000 | **-** | - | 0.0000 | **-** | - |
| **Skin and soft tissue infection** |  |  |  |  |  |  |
| Intercept β_0_ | -8.1529 | -8.4673,-7.8385 | <.0001 | -8.3357 | -8.4016,-8.2698 | <.0001 |
| Baseline trend β_1_ | -0.0040 | -0.0088,0.0007 | 0.0961 | - | - | - |
| SARS β_2_ | 0.2258 | -0.0432,0.4949 | 0.1000 | - | - | - |
| Level change after hand hygiene β_3_ | 0.1793 | -0.0662,0.4248 | 0.1522 | - | - | - |
| Trend change after hand hygiene β_4_ | -0.0052 | -0.0138,0.0034 | 0.2352 | -0.0084 | -0.0125,-0.0042 | <.0001 |
| Season |  |  | 0.4620 |  |  | - |
| Season1 (Mar-May) β_5_ | -0.0116 | -0.1742,0.1510 | 0.8890 | - | - | - |
| Season2 (Jun-Aug) β_6_ | -0.0848 | -0.2528,0.0831 | 0.3220 | - | - | - |
| Season3 (Sept-Nov) β_7_ | -0.1087 | -0.2713,0.0540 | 0.1904 | - | - | - |
| Season4 (Dec-Feb) | 0.0000 | **-** | - | - | - | - |
| **Surgical site infection** |  |  |  |  |  |  |
| Intercept β_0_ | -6.4209 | -6.7511,-6.0907 | <.0001 | -6.6685 | -6.7367,-6.6004 | <.0001 |
| Baseline trend β_1_ | -0.0042 | -0.0072,-0.0012 | 0.0063 | -0.0055 | -0.0067,-0.0044 | <.0001 |
| SARS β_2_ | 0.1251 | -0.0419,0.2921 | 0.1422 | 0.2340 | 0.0936,0.3745 | 0.0011 |
| Level change after hand hygiene β_3_ | -0.0966 | -0.2568,0.0636 | 0.2374 | - | - | - |
| Trend change after hand hygiene β_4_ | -0.0032 | -0.0086,0.0021 | 0.2327 | - | - | - |
| Season |  |  | 0.3680 |  |  | - |
| Season1 (Mar-May) β_5_ | 0.0140 | -0.0943,0.1224 | 0.7997 | - | - | - |
| Season2 (Jun-Aug) β_6_ | 0.0856 | -0.0218,0.1929 | 0.1181 | - | - | - |
| Season3 (Sept-Nov) β_7_ | 0.0417 | -0.0650,0.1485 | 0.4435 | - | - | - |
| Season4 (Dec-Feb) | 0.0000 | **-** | - | - | - | - |
| **Respiratory tract infection** |  |  |  |  |  |  |
| Intercept β_0_ | -7.5701 | -7.8226,-7.3175 | <.0001 | -7.5405 | -7.5762,-7.5048 | <.0001 |
| Baseline trend β_1_ | 0.0004 | -0.0028,0.0036 | 0.8040 | - | - | - |
| SARS β_2_ | 0.0903 | -0.0825,0.2631 | 0.3055 | - | - | - |
| Level change after hand hygiene β_3_ | -0.0525 | -0.2109,0.1059 | 0.5161 | - | - | - |
| Trend change after hand hygiene β_4_ | -0.0010 | -0.0063,0.0044 | 0.7253 | - | - | - |
| Season |  |  | 0.1732 |  |  | - |
| Season1 (Mar-May) β_5_ | 0.0951 | -0.0116,0.2018 | 0.0806 | - | - | - |
| Season2 (Jun-Aug) β_6_ | 0.0125 | -0.0956,0.1205 | 0.8211 | - | - | - |
| Season3 (Sept-Nov) β_7_ | -0.0064 | -0.1121,0.0992 | 0.9048 | - | - | - |
| Season4 (Dec-Feb) | 0.0000 | **-** | - | - | - | - |
| **Gastrointestinal infection** |  |  |  |  |  |  |
| Intercept β_0_ | -10.4557 | -10.9093,-10.0022 | <.0001 | -10.2424 | -10.4945,-9.9903 | <.0001 |
| Baseline trend β_1_ | 0.0100 | 0.0001,0.0198 | 0.0480 | 0.0057 | 0.0019,0.0095 | 0.0032 |
| SARS β_2_ | -0.6805 | -1.3407,-0.0202 | 0.0434 | - | - | - |
| Level change after hand hygiene β_3_ | -0.0480 | -0.5026,0.4066 | 0.8360 | - | - | - |
| Trend change after hand hygiene β_4_ | -0.0076 | -0.0221,0.0068 | 0.3012 | - | - | - |
| Number of infection at time lag1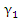 | 0.0494 | 0.0099,0.0889 | 0.0142 | 0.0650 | 0.0289,0.1011 | 0.0004 |
| Season |  |  | 0.0363 |  |  | - |
| Season1 (Mar-May) β_5_ | 0.3566 | 0.0552,0.6579 | 0.0204 | - | - | - |
| Season2 (Jun-Aug) β_6_ | 0.1769 | -0.1344,0.4882 | 0.2653 | - | - | - |
| Season3 (Sept-Nov) β_7_ | -0.0278 | -0.3452,0.2896 | 0.8637 | - | - | - |
| Season4 (Dec-Feb) | 0.0000 | **-** | - | - | - | - |
| ***Staphylococcus aureus* infection** |  |  |  |  |  |  |
| Intercept β_0_ | -7.3461 | -7.5669,-7.1253 | <.0001 | -7.3878 | -7.5489,-7.2268 | <.0001 |
| Baseline trend β_1_ | 0.0003 | -0.0024,0.0029 | 0.8362 | - | - | - |
| SARS β_2_ | -0.0214 | -0.1722,0.1294 | 0.7813 | - | - | - |
| Level change after hand hygiene β_3_ | -0.1523 | -0.2902,-0.0144 | 0.0304 | -0.2122 | -0.2774,-0.1470 | <.0001 |
| Trend change after hand hygiene β_4_ | -0.0035 | -0.0084,0.0013 | 0.1560 | - | - | - |
| Number of infection at time lag1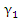 | 0.0045 | 0.0001,0.0088 | 0.0440 | 0.0060 | 0.0020,0.0100 | 0.0033 |
| Season |  |  | 0.2787 |  |  | - |
| Season1 (Mar-May) β_5_ | 0.0242 | -0.0674,0.1157 | 0.6050 | - | - | - |
| Season2 (Jun-Aug) β_6_ | -0.0408 | -0.1341,0.0525 | 0.3909 | - | - | - |
| Season3 (Sept-Nov) β_7_ | -0.0534 | -0.1437,0.0369 | 0.2462 | - | - | - |
| Season4 (Dec-Feb) | 0.0000 | **-** | - | - | - | - |
| **Methicillin-resistant *S. aureus* infection** | |  |  |  |  |  |
| Intercept β_0_ | -7.7245 | -7.9460,-7.5031 | <.0001 | -7.7035 | -7.8640,-7.5431 | <.0001 |
| Baseline trend β_1_ | 0.0014 | -0.0017,0.0045 | 0.3761 | - | - | - |
| SARS β_2_ | -0.0700 | -0.2425,0.1025 | 0.4265 | - | - | - |
| Level change after hand hygiene β_3_ | -0.1948 | -0.3566,-0.0330 | 0.0183 | -0.1364 | -0.2604,-0.0125 | 0.0309 |
| Trend change after hand hygiene β_4_ | -0.0061 | -0.0118,-0.0004 | 0.0359 | -0.0049 | -0.0095,-0.0003 | 0.0373 |
| Number of infection at time lag1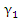 | 0.0070 | 0.0013,0.0126 | 0.0151 | 0.0087 | 0.0035,0.0140 | 0.0012 |
| Season |  |  | 0.0444 |  |  | - |
| Season1 (Mar-May) β_5_ | 0.1057 | -0.0012,0.2126 | 0.0525 | - | - | - |
| Season2 (Jun-Aug) β_6_ | -0.0053 | -0.1153,0.1047 | 0.9246 | - | - | - |
| Season3 (Sept-Nov) β_7_ | -0.0303 | -0.1373,0.0767 | 0.5786 | - | - | - |
| Season4 (Dec-Feb) | 0.0000 | **-** | - | - | - | - |
| **Methicillin-susceptible *S. aureus* infection** | |  |  |  |  |  |
| Intercept β_0_ | -8.4455 | -8.7960,-8.0950 | <.0001 | -8.4722 | -8.5939,-8.3506 | <.0001 |
| Baseline trend β_1_ | -0.0035 | -0.0086,0.0017 | 0.1856 | -0.0020 | -0.0039,-0.0000 | 0.0451 |
| SARS β_2_ | 0.1217 | -0.1811,0.4244 | 0.4309 | - | - | - |
| Level change after hand hygiene β_3_ | -0.0446 | -0.3177,0.2285 | 0.7488 | - | - | - |
| Trend change after hand hygiene β_4_ | 0.0053 | -0.0035,0.0140 | 0.2385 | - | - | - |
| Season |  |  | 0.2375 |  |  | - |
| Season1 (Mar-May) β_5_ | -0.1799 | -0.3608,0.0010 | 0.0512 | - | - | - |
| Season2 (Jun-Aug) β_6_ | -0.1250 | -0.3050,0.0550 | 0.1734 | - | - | - |
| Season3 (Sept-Nov) β_7_ | -0.0608 | -0.2332,0.1116 | 0.4895 | - | - | - |
| Season4 (Dec-Feb) | 0.0000 | **-** | **-** | - | - | - |
| ***Acinetobacter* infection** |  |  |  |  |  |  |
| Intercept β_0_ | -8.2063 | -8.4177,-7.9950 | <.0001 | -8.0570 | -8.1905,-7.9236 | <.0001 |
| Baseline trend β_1_ | 0.0032 | -0.0008,0.0072 | 0.1163 | 0.0063 | 0.0037,0.0088 | <.0001 |
| SARS β_2_ | 0.2649 | 0.0887,0.4410 | 0.0032 | 0.2470 | 0.0908,0.4031 | 0.0019 |
| Level change after hand hygiene β_3_ | -0.0421 | -0.2107,0.1265 | 0.6247 | - | - | - |
| Trend change after hand hygiene β_4_ | -0.0042 | -0.0104,0.0019 | 0.1734 | -0.0093 | -0.0147,-0.0039 | 0.0008 |
| Season |  |  | 0.0147 |  |  | 0.0007 |
| Season1 (Mar-May) β_5_ | 0.0065 | -0.1191,0.1321 | 0.9191 | -0.0211 | -0.1382,0.0960 | 0.7244 |
| Season2 (Jun-Aug) β_6_ | 0.1474 | 0.0308,0.2640 | 0.0132 | 0.1477 | 0.0349,0.2605 | 0.0103 |
| Season3 (Sept-Nov) β_7_ | 0.1236 | 0.0107,0.2364 | 0.0319 | 0.1568 | 0.0457,0.2679 | 0.0057 |
| Season4 (Dec-Feb) | 0.0000 | **-** | - | 0.0000 | **-** | - |
| **Extensively drug-resistant *A. baumannii* infection** | | |  |  |  |  |
| Intercept β_0_ | -10.8453 | -11.2272,-10.4634 | <.0001 | -10.7734 | -11.0758,-10.4711 | <.0001 |
| Baseline trend β_1_ | 0.0417 | 0.0319,0.0515 | <.0001 | 0.0431 | 0.0374,0.0488 | <.0001 |
| SARS β_2_ | 0.0726 | -0.2074,0.3527 | 0.6112 | - | - | - |
| Level change after hand hygiene β_3_ | -0.0409 | -0.3271,0.2453 | 0.7796 | - | - | - |
| Trend change after hand hygiene β_4_ | -0.0588 | -0.0745,-0.0431 | <.0001 | -0.0618 | -0.0713,-0.0523 | <.0001 |
| Season |  |  | 0.3444 |  |  | - |
| Season1 (Mar-May) β_5_ | 0.1613 | -0.0382,0.3608 | 0.1130 | - | - | - |
| Season2 (Jun-Aug) β_6_ | 0.1681 | -0.0345,0.3707 | 0.1039 | - | - | - |
| Season3 (Sept-Nov) β_7_ | 0.0915 | -0.1039,0.2869 | 0.3588 | - | - | - |
| Season4 (Dec-Feb) | 0.0000 | - | - | - | - | - |
| ***Escherichia coli* infection** |  |  |  |  |  |  |
| Intercept β_0_ | -7.6854 | -7.8682,-7.5026 | <.0001 | -7.6605 | -7.7843,-7.5368 | <.0001 |
| Baseline trend β_1_ | 0.0018 | -0.0013,0.0049 | 0.2573 | 0.0033 | 0.0017,0.0048 | <.0001 |
| SARS β_2_ | 0.1119 | -0.0414,0.2651 | 0.1525 | - | - | - |
| Level change after hand hygiene β_3_ | 0.0092 | -0.1283,0.1468 | 0.8951 | - | - | - |
| Trend change after hand hygiene β_4_ | 0.0022 | -0.0022,0.0065 | 0.3313 | - | - | - |
| Number of infection at time lag1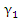 | 0.0064 | 0.0019,0.0109 | 0.0054 | 0.0060 | 0.0017,0.0104 | 0.0063 |
| Season |  |  | 0.2435 |  |  | - |
| Season1 (Mar-May) β_5_ | 0.0668 | -0.0248,0.1583 | 0.1530 | - | - | - |
| Season2 (Jun-Aug) β_6_ | 0.0015 | -0.0913,0.0942 | 0.9755 | - | - | - |
| Season3 (Sept-Nov) β_7_ | -0.0195 | -0.1102,0.0712 | 0.6740 | - | - | - |
| Season4 (Dec-Feb) | 0.0000 | - | - | - | - | - |
| **Intensive care units** |  |  |  |  |  |  |
| Intercept β_0_ | -4.4032 | -4.5641,-4.2422 | <.0001 | -4.2936 | -4.3620,-4.2253 | <.0001 |
| Baseline trend β_1_ | 0.0018 | -0.0004,0.0041 | 0.1110 | 0.0021 | 0.0008,0.0034 | 0.0016 |
| SARS β_2_ | 0.0522 | -0.0477,0.1521 | 0.3059 | - | - | - |
| Level change after hand hygiene β_3_ | -0.0529 | -0.1449,0.0391 | 0.2600 | - | - | - |
| Trend change after hand hygiene β_4_ | -0.0060 | -0.0101,-0.0019 | 0.0038 | -0.0080 | -0.0108,-0.0053 | <.0001 |
| Season |  |  | <.0001 |  |  | <.0001 |
| Season1 (Mar-May) β_5_ | 0.1449 | 0.0810,0.2089 | <.0001 | 0.1343 | 0.0740,0.1946 | <.0001 |
| Season2 (Jun-Aug) β_6_ | 0.1175 | 0.0533,0.1818 | 0.0003 | 0.1107 | 0.0499,0.1715 | 0.0004 |
| Season3 (Sept-Nov) β_7_ | 0.1419 | 0.0786,0.2052 | <.0001 | 0.1437 | 0.0836,0.2037 | <.0001 |
| Season4 (Dec-Feb) | 0.0000 | **-** | - | 0.0000 | **-** | - |
| **Hematology ward** |  |  |  |  |  |  |
| Intercept β_0_ | -4.4273 | -4.6882,-4.1665 | <.0001 | -4.4452 | -4.5723,-4.3181 | <.0001 |
| Baseline trend β_1_ | 0.0040 | -0.0000,0.0081 | 0.0518 | 0.0032 | 0.0017,0.0046 | <.0001 |
| SARS β_2_ | -0.1577 | -0.4236,0.1082 | 0.2451 | - | - | - |
| Level change after hand hygiene β_3_ | -0.1312 | -0.3358,0.0734 | 0.2087 | - | - | - |
| Trend change after hand hygiene β_4_ | 0.0029 | -0.0036,0.0094 | 0.3770 | - | - | - |
| Season |  |  | 0.0114 |  |  | 0.0166 |
| Season1 (Mar-May) β_5_ | 0.1226 | -0.0182,0.2635 | 0.0880 | 0.0963 | -0.0375,0.2300 | 0.1583 |
| Season2 (Jun-Aug) β_6_ | 0.2144 | 0.0748,0.3541 | 0.0026 | 0.1929 | 0.0608,0.3249 | 0.0042 |
| Season3 (Sept-Nov) β_7_ | 0.1968 | 0.0601,0.3335 | 0.0048 | 0.1766 | 0.0457,0.3075 | 0.0082 |
| Season4 (Dec-Feb) | 0.0000 | - | - | 0.0000 | - | - |
| **Oncology wards** |  |  |  |  |  |  |
| Intercept β_0_ | -4.8143 | -5.0049,-4.6238 | <.0001 | -4.8333 | -4.9453,-4.7213 | <.0001 |
| Baseline trend β_1_ | 0.0008 | -0.0030,0.0047 | 0.6732 | 0.0021 | 0.0008,0.0034 | 0.0010 |
| SARS β_2_ | 0.1665 | -0.0591,0.3920 | 0.1480 | - | - | - |
| Level change after hand hygiene β_3_ | 0.0570 | -0.1181,0.2320 | 0.5234 | - | - | - |
| Trend change after hand hygiene β_4_ | -0.0006 | -0.0061,0.0049 | 0.8354 | - | - | - |
| Season |  |  | 0.0242 |  |  | 0.0010 |
| Season1 (Mar-May) β_5_ | 0.1015 | -0.0174,0.2204 | 0.0942 | 0.1561 | 0.0429,0.2694 | 0.0069 |
| Season2 (Jun-Aug) β_6_ | 0.1519 | 0.0319,0.2719 | 0.0131 | 0.2006 | 0.0860,0.3152 | 0.0006 |
| Season3 (Sept-Nov) β_7_ | 0.1664 | 0.0525,0.2804 | 0.0042 | 0.2075 | 0.0957,0.3193 | 0.0003 |
| Season4 (Dec-Feb) | 0.0000 | - | - | 0.0000 | - | - |
| **Other wards** |  |  |  |  |  |  |
| Intercept β_0_ | -5.7538 | -5.8849,-5.6227 | <.0001 | -5.7427 | -5.8349,-5.6504 | <.0001 |
| Baseline trend β_1_ | 0.0018 | 0.0003,0.0033 | 0.0162 | 0.0009 | 0.0003,0.0015 | 0.0040 |
| SARS β_2_ | 0.1105 | 0.0432,0.1778 | 0.0013 | 0.1380 | 0.0821,0.1939 | <.0001 |
| Level change after hand hygiene β_3_ | -0.0403 | -0.1011,0.0205 | 0.1937 | - | - | - |
| Trend change after hand hygiene β_4_ | -0.0008 | -0.0030,0.0013 | 0.4359 | - | - | - |
| Number of infection at time lag1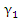 | 0.0011 | 0.0005,0.0018 | 0.0004 | 0.0012 | 0.0006,0.0018 | <.0001 |
| Season |  |  | 0.8726 |  |  | - |
| Season1 (Mar-May) β_5_ | 0.0021 | -0.0406,0.0447 | 0.9242 | - | - | - |
| Season2 (Jun-Aug) β_6_ | -0.0019 | -0.0436,0.0398 | 0.9278 | - | - | - |
| Season3 (Sept-Nov) β_7_ | -0.0136 | -0.0540,0.0268 | 0.5090 | - | - | - |
| Season4 (Dec-Feb) | 0.0000 | - | - | - | - | - |
