# Supplementary material for: Effectiveness and Limitations of Hand Hygiene Promotion on Decreasing Healthcare–Associated Infections
Source: PLoS One. 2011 Nov 16;6(11):e27163. doi: 10.1371/journal.pone.0027163 (PMC3217962; doi:10.1371/journal.pone.0027163)
Supplement: Table S2 — Base-case estimates and ranges used in sensitivity analyses of parameters. (DOC) [file pone.0027163.s003.doc]

**Table S2** Base-case estimates and ranges used in sensitivity analyses of parameters

| **Variables** | **Base-Case Estimate** | **Range** | **Sources** |
| --- | --- | --- | --- |
| 1. Costs |  |  |  |
| Alcohol handrub | 221,517 | 110,759~332,276 | Current Study |
| Campaign | 22,953 | 11,477~34,430 | Current Study |
| Personnel ^a^ | 0 | 0~329,000 | Current Study |
| 1. Benefit |  |  |  |
| Extra cost per episode of  healthcare-associated  infection | 3,877 | -390~11,502 | [18] |
| 1. Discount rate | 3% | 0%~7% | -- |

^a^ The cost of the personnel involving in the program including planning and training was not considered in the base-case analysis, because no increase in manpower due to the program and no staff were paid by the program.
