# Supplementary material for: Effectiveness and Limitations of Hand Hygiene Promotion on Decreasing Healthcare–Associated Infections
Source: PLoS One. 2011 Nov 16;6(11):e27163. doi: 10.1371/journal.pone.0027163 (PMC3217962; doi:10.1371/journal.pone.0027163)
Supplement: Table S3 — Results of one-way sensitivity analysis. (DOC) [file pone.0027163.s004.doc]

**Table S3** Results of one-way sensitivity analysis

| **Variables** | **Incremental Cost-effectiveness Ratio** ^a^ | **Cost-benefit analysis** | |
| --- | --- | --- | --- |
|  |  | Net Benefit ^b^ | Benefit Cost Ratio ^c^ |
| Base-case | 163.6 | 5,289,364 | 23.7 |
| Alcohol handrub costs | 89.5-237.7 | 5,394,958-5,183,770 | 43.3-16.3 |
| Campaign expense | 155.9-171.3 | 5,300,292-5,278,436 | 24.9-22.6 |
| Personnel cost ^d^ | 163.6-220.4 | 5,289,364-5,208,486 | 23.7-17.6 |
| Extra cost per episode of HAI | 163.6-163.6 | -787,849-16,150,717 | -2.4-70.3 |
| Averted episodes of HAI ^e^ | 468.3-96.7 | 1,696,226-9,112,938 | 8.3-40.1 |
| Discount rate | 162.6-164.9 | 5,585,638-4,937,617 | 23.8-23.5 |

^a^ Extra cost required to prevent one episode of healthcare-associated infection (HAI).

^b^ Net benefit= Benefit – Cost.

^c^ Benefit cost ratio=benefit/cost.

^d^ The opportunity cost of personnel were considered in the sensitivity analysis by converting the working hours spent on the program by the infection control nurses according to their salary.

^e^ The range of averted episodes of HAI were 526-2,544.
